# Recurrent Mental Status Changes in a Patient With Chronic Alcoholic Cirrhosis Taking Diuretics: A Case Report

**DOI:** 10.7759/cureus.48221

**Published:** 2023-11-03

**Authors:** Emily A Colalillo, Peter J Rogu, Jonathan Wierzbicki

**Affiliations:** 1 Medicine, New York Institute of Technology (NYIT) College of Osteopathic Medicine, Old Westbury, USA; 2 Family Medicine, Hunterdon Medical Center, Flemington, USA

**Keywords:** alcohol-related cirrhosis, thiazide diuretics, serum ammonia, diuretic induced, cirrhosis of the liver, chronic liver disease, hepatic encephalopathy (he)

## Abstract

Cirrhosis of the liver, characterized by fibrous tissue replacing normal cells, disrupts physiological function and blood circulation. A further consequence of this is hepatic encephalopathy (HE), a neuropsychiatric syndrome that can range in severity from mild cognitive disturbances to full coma. This case follows the course of a 63-year-old Caucasian female with chronic liver cirrhosis who presents with recurrent episodes of mental status changes. Although each episode was treated with first-line pharmacologic interventions of lactulose, her HE recurrence persisted. This case report underscores the significance of early diagnosis and management, emphasizing the role of alcohol cessation, pharmacotherapy, and lifestyle adjustments. It also aims to address the delicate balance of diuretic use, focusing on dosage adjustments to address electrolyte imbalances and minimize risks associated with HE. The findings highlight the complexity of managing alcoholic liver disease and offer insights into tailored approaches for optimizing patient outcomes.

## Introduction

Liver cirrhosis represents a severe manifestation of alcoholic liver disease, which can result in fatalities and unfortunate health complications. Cirrhosis is characterized by fibrous scar tissue replacing the normal hepatic tissue, impeding physiologic function. Epidemiological reports suggest that roughly 10-15% of individuals with chronic alcoholism eventually develop this condition [1]. Cirrhosis presents clinically with jaundice, palmar erythema, ascites, spider angiomas, anorexia, and fatigue [2]. On diagnostic tests, there may be an elevation in bilirubin, aspartate aminotransferase (AST), and alanine transaminase (ALT) while hemoglobin, albumin, and platelet counts become low [3]. Initial treatment involves immediate alcohol cessation. Newer options such as naltrexone and acamprosate have demonstrated effectiveness in diminishing and potentially eradicating alcohol intake among individuals with a history of chronic heavy drinking [4]. Other lifestyle modifications such as smoking cessation are imperative as smoking can lead to the progression of hepatocellular carcinoma [5]. 

A further consequence of cirrhosis is the development of hepatic encephalopathy (HE), a complex neuropsychiatric syndrome. HE manifests a range of neurological symptoms, from subtle cognitive disturbances and asterixis to profound coma. The underlying pathophysiology is intricate and not fully understood; however, leading theories propose that the increased blood levels of ammonia products by enteric bacteria play a pivotal role. The normal metabolism of ammonia is dependent on hepatocytes, with failure leading to its accumulation [6]. Alternatively, portal hypertension may lead to portosystemic shunting and a loss of first-pass metabolism of ammonia [7]. The elevated ammonia can lead to osmotic gradient-induced cerebral edema and/or directly impact neuronal transmission [6,8]. Treatment primarily aims to reduce the nitrogenous load produced by enteric bacteria and lower systemic ammonia levels. This is commonly achieved by using lactulose as the first-line medication. In addition, rifaximin, L-ornithine L-aspartate, and more drastically, surgical excision of the colon, occlusion of portosystemic collaterals, and liver transplant can be considered [9,10]. 

Neurologic symptoms related to chronic alcohol consumption are not exclusively linked to HE. Another well-documented neuropsychiatric illness is Wernicke’s encephalopathy (WE), a result of vitamin B1 deficiency commonly seen in heavy drinkers. The classic triad of symptoms is mental status change, ataxia, and oculomotor abnormality; however, only 16% of patients present as such and 19% of patients have no clinical signs [11,12]. Timely thiamine administration can reverse the symptoms and prevent long-term complications [13]. Chronic alcohol consumption can also destroy brain cells, contract brain tissue, and depress the central nervous system by suppressing excitatory nerve pathways. This ultimately leads to neurodegeneration, memory impairment, and cognitive decline [14]. 

Within the context of this specific case presentation, our attention will be directed toward the examination of HE secondary to alcohol-induced cirrhosis and the sudden onset of severe hepatobiliary and neurologic symptoms.

## Case presentation

A 63-year-old Caucasian female with a history of alcoholic liver cirrhosis, portal hypertension, hypertension, chronic kidney disease, depression, and anemia presented to the emergency department on multiple occasions during March of 2023. The primary concern prompting these visits was a notable alteration in cognitive function. As per accounts provided by the patient's significant other, the patient’s health state exhibited a progressive decline over the course of the month, necessitating ambulance services every other day. The patient was described as weak, incoherent, and experienced significant changes in her mental status. The patient had been followed regularly by her primary care physician, and most recent laboratory results show an array of abnormalities as listed in Table 1. Most pertinently, her ammonia levels and liver function tests were elevated in a manner suggestive of alcoholic liver cirrhosis induced HE. There were additional electrolyte imbalances as well as hematological and renal concerns. Despite these detrimental changes, the informant attested to faithful adherence of the patient to the prescribed medication regimen, as outlined in Table 2. The only inconsistency noted in her medication record from her previous health check with her family physician in June of 2022 was the addition of hydrochlorothiazide at an undocumented date.

**Table 1:** Patient's Lab Values From August 2022 — Prior to Hospital Admission Flags: H: High, N: Normal, L: Low; BUN: blood urea nitrogen; eGFR: estimated glomerular filtration rate; MCV: mean corpuscular volume; MCH: mean corpuscular hemoglobin; MCHC: mean corpuscular hemoglobin concentration; RDW: red cell distribution width; AST: aspartate aminotransferase; ALT: alanine aminotransferase

Description	Result	Range	Flag
Ammonia, Plasma	442 ug/dL	34-178 ug/dL	H
Basic Metabolic Panel			
Glucose	106 mg/dL	65-99 mg/dL	H
BUN	63 mg/dL	8-27 mg/dL	H
Creatinine	2.53 mg/dL	0.57-1.00 mg/dL	H
eGFR	21 mL/min/1.73	>59 mL/min/1.73	L
BUN/Creatinine Ratio	25	12-28	N
Sodium	131 mmol/L	134-144 mmol/L	L
Potassium	5.6 mmol/L	3.5-5.2 mmol/L	H
Chloride	98 mmol/L	96-106 mmol/L	N
Carbon Dioxide, Total	18 mmol/L	20-29 mmol/L	L
Calcium	9.4 mg/dL	8.7-10.3 mg/dL	N
CBC w/ Platelet, no differential			
wBC	5.5 x10E3/uL	34-108 x10E3/uL	N
RBC	2.67 x10E6/uL	3.77-5.28 x10E6/uL	L
Hemoglobin	8.3 g/dL	11.1-15.9 g/dL	L
Hematocrit	24.9 %	34.0-46.6 %	L
MCV	93 fL	79-97 fL	N
MCH	31.1 pg	26.6-33.0 pg	N
MCHC	33.3 g/dL	31.5-35.7 g/dL	N
RDW	14.4 %	11.7-15.4 %	N
Platelets	140 x10E3/uL	150-450 x10E3/uL	L
Hepatic Function Test			
Protein, Total	6.9 g/dL	6.0-8.5 g/dL	N
Albumin	3.5 g/dL	3.8-48 g/dL	L
Bilirubin, Total	1 mg/dL	0.0-1.2 mg/dL	N
Bilirubin, Direct	0.47 mg/dL	0.00-0.40 mg/dL	H
Alkaline Phosphatase	129 IU/L	44-121 IU/L	H
AST (SGOT)	65 IU/L	0-40 IU/L	H
ALT (SGPT)	32 IU/L	0-32 IU/L	N
Uric Acid			
Uric Acid	10 mg/dL	3.0-7.2 mg/dL	H

**Table 2:** Patient's Medication List from March 2023 — Prior to Her Hospital Admissions

Medication Name	Directions
Lactulose 10 g/15 mL oral solution	Take two teaspoonfuls by mouth three times per day as needed
Ferrous sulfate 325 mg (65 mg iron) tablet	Take one tablet orally two times per day
Furosemide 40 mg tablet	Take one tablet by mouth every day
Hydrochlorothiazide 12.5 mg tablet	Take one tablet by mouth every day
Hydroxyzine HCl 25 mg tablet	Take one tablet by mouth every day as needed
Mucinex 600 mg tablet, extended release	Take one tablet orally every twelve hours as needed
Nadolol 20 mg tablet	Take one tablet by mouth every day
Ondansetron HCl 4 mg tablet	Take one tablet by mouth three times per day as needed
Pantoprazole 40 mg tablet, delayed release	Take one tablet by mouth every day
Prochlorperazine Maleate 10 mg tablet	Take one tablet by mouth three times per day as needed
Spironolactone 100 mg tablet	Take one tablet by mouth every day
Thiamine Hcl 100 mg tablet	Take one tablet by mouth every day
Trazodone 50 mg tablet	Take one tablet by mouth every day after meals
Vitamin B-12 1,000 mcg tablet	Take one tablet by mouth every day
Xifaxan 550 mg tablet	Take one tablet by mouth twice per day

The patient was found to have recurrent HE. At the time of her most recent hospital admission, her ammonia level was again severely elevated. She was put in physical restraints and had a nasogastric tube placed to receive lactulose at an increased dose of 20mg/30mL three times per day. An ultrasound of the abdomen was also conducted and showed a heterogeneous coarsened echotexture and a nodular contour consistent with cirrhosis. A CT of the head without contrast was further ordered. It showed no acute intracranial hemorrhage or mass effect. Noted were chronic small vessel and white matter changes as well as a chronic right cerebellar lacunar infarct. 

Upon improvement of her mental status, the nasogastric tube was removed, and she tolerated a diet well. Electrolyte imbalances were corrected, as she was found to have severe hypomagnesemia. The patient also had a GI consult done, which concluded that the suspect etiology of HE was related to dietary indiscretions and medication dosages. Upon discharge from the hospital, the patient was instructed to continue with the lactulose 20 mg/30 mL oral solution three times per day, have adequate hydration, begin magnesium supplements, and have a goal of 2-3 bowel movements per day. Her hospital chart also included a medication list to be followed upon returning home, which included the addition of magnesium oxide, the removal of hydrochlorothiazide, a decrease in the dose of furosemide to 20 mg/day, and a decrease in the dose of spironolactone to 25 mg/day.

## Discussion

HE is a significant neurological disorder that manifests in profound liver dysfunction, predominantly in advanced liver cirrhosis. The pathophysiology of HE is rooted in a multifaceted interplay of biochemical and neurophysiological processes. As delineated in Table 3, several precipitating factors can lead to episodic and recurrent HE, with some being more prevalent than others [15].

**Table 3:** Precipitating Factors of Episodic and Recurrent Hepatic Encephalopathy in Descending Order

Episodic hepatic encephalopathy	Recurrent hepatic encephalopathy
Infections	Electrolyte abnormalities
Gastrointestinal bleeding	Infections
Diuretics	Unidentified
Electrolyte abnormalities	Constipation
Constipation	Diuretic overdose
Unidentified	Gastrointestinal bleeding

Electrolyte imbalance stands out as the foremost cause of recurrent HE [15]. During the patient's hospitalization, she exhibited markedly elevated ammonia levels. Elevated serum ammonia plays a pivotal role in altering brain function, impeding neurotransmission, and thereby results in a spectrum of neurological manifestations [6]. To address the patient’s recurrent HE, pharmacological modifications were instituted. Her lactulose dosage doubled from 10 g/15 mL to 20 g/30 mL oral solution. Lactulose is a non-absorbable, synthetic, disaccharide composed of galactose and fructose. It mitigates ammonia levels through several mechanisms. Firstly, lactulose metabolism in the colon increases osmolarity and produces intraluminal gas, leading to a laxative effect and reduced colon transit time, thereby narrowing the absorption window for various substances. Secondly, lactulose fosters an acidic colonic environment, converting ammonia (NH3) to ammonium (NH4+), which, due to its positive charge, cannot diffuse across biological membranes. Lastly, the produced acidic environment is uninhabitable by ammonia-producing bacteria, reducing their numbers [16].

The patient's clinical profile also revealed pronounced hypomagnesemia, a recognized adverse effect of the diuretic furosemide [17]. Furosemide functions by inhibiting the sodium-chloride cotransport channel in the thick ascending limb of the loop of Henle. This action amplifies the excretion of water, sodium, chloride, magnesium, and calcium in that segment [17]. In patients with underlying liver conditions, careful use of furosemide is imperative due to the heightened risk of rapid electrolyte shifts, potentially precipitating HE [17]. This rationale can explain the decision to reduce the patient's Furosemide dosage from 40 mg/day to 20 mg/day.

Concurrently, the spironolactone dosage was adjusted from 100 mg/day to 25 mg/day. Spironolactone, a diuretic, antagonizes mineralocorticoid receptors, particularly those binding to aldosterone. Under physiological conditions, aldosterone maintains sodium and potassium homeostasis, by reabsorbing sodium and excreting potassium. By inhibiting aldosterone, spironolactone reduces sodium reabsorption and promotes potassium retention in the renal tubules [18]. Notably, in isolated instances, spironolactone has been implicated in exacerbating liver injury, which might have contributed to the patient's recurrent HE and necessitated the dosage reduction [18].

Lastly, the complete discontinuation of hydrochlorothiazide was deemed necessary. As a thiazide diuretic, hydrochlorothiazide can greatly precipitate electrolyte disturbances. Regular monitoring for electrolyte anomalies, such as hypomagnesemia, is essential when prescribing this medication, especially in the backdrop of liver dysfunction. Given the inherent risks, thiazides should be prescribed with caution in patients with such a condition, to avoid the exacerbation of HE [19]. The decision to cease Hydrochlorothiazide reflects an intent to streamline her diuretic regimen and stabilize her electrolyte profile.

While brain lesions and tumors, strokes, hemorrhages, CNS infections, and seizures may also present with an altered mental status, neuroimaging was appropriate to rule out possible differentials. The CT findings of the head were not significant. Although it showed chronic small vessel and white matter changes as well as a chronic right cerebellar lacunar infarct, those abnormalities would not present with recurrent and acute episodes of mental status changes. Due to her past medical history, current clinical presentation, laboratory values, a negative CT scan, and significant improvement with traditional HE treatment, other diagnostic tests such as EEG and brain MRI were not performed due to a strong suspicion of HE as the primary differential.

## Conclusions

There are many intricate dynamics, as shown in this case, when treating HE within the context of advanced liver cirrhosis. The patient's clinical presentation, marked by recurrent episodes of altered mental status, was closely linked to elevated ammonia levels, emphasizing the pivotal role of electrolyte imbalance in HE. Pharmacological interventions, including doubling lactulose dosage for its multifaceted ammonia-reducing mechanisms, and judicious adjustments to diuretic medications aimed at mitigating rapid electrolyte shifts, formed the cornerstone of treatment. Although the diagnosis of HE seemed apparent, it was still crucial to rule out other causes of altered mental status with the use of neuroimaging.

This case illustrates the multifaceted nature of alcoholic liver disease, its consequences, and the intricate management required to mitigate associated risks. Individualized treatment strategies, vigilant medication adjustments, and ongoing monitoring are crucial to enhancing patients' quality of life and preventing further complications, especially with concurrent use of pharmacological diuretics.
